# Prognostic and Clinicopathological Value of Survivin in Diffuse Large B-cell Lymphoma: A Meta-Analysis

**DOI:** 10.1097/MD.0000000000001432

**Published:** 2015-09-11

**Authors:** Ya Zhang, Jianhong Wang, Xiaohui Sui, Ying Li, Kang Lu, Xiaosheng Fang, Yujie Jiang, Xin Wang

**Affiliations:** From the Department of Hematology, Shandong Provincial Hospital affiliated to Shandong University (YZ, JW, XS, YL, KL, XF, YJ, XW); and Institute of Diagnostics, Shandong University School of Medicine, Jinan, Shandong, P.R. China (XW).

## Abstract

Up to date, survivin, a well-known inhibitor of apoptosis, has attracted considerable attention as a potential biomarker and therapeutic target in diffuse large B-cell lymphoma (DLBCL). Nevertheless, there still remains no consensus on heterogeneous results. Herein, a meta-analysis was performed to clarify a convincing significance of survivin status on prognosis and clinicopathology of DLBCL patients.

Eligible studies were identified by searching Medline, Embase, Scopus, CNKI, and Wanfang databases (last updated on November 30, 2014). Pooled hazard ratios (HRs) and odds ratios (ORs) with 95% confidence intervals (CIs) were calculated. Heterogeneity and sensitivity were also analyzed. Moreover, Begg, Egger test, and funnel plots were applied to evaluate the publication bias.

We finally included 17 eligible studies with the total number of 1352 patients in the meta-analysis. The pooled results showed that positive survivin expression in DLBCL was associated with inferior overall survival (OS) (HR: 1.880, 95% CI: 1.550–2.270) in patients. Moreover, a significant association was revealed between survivin expression and advanced clinical stage (III + IV) (OR: 0.611, 95% CI: 0.452–0.827), higher International Prognosis Index (IPI) score (Score 3–5) (OR: 0.559; 95% CI: 0.410–0.761), elevated serum lactic dehydrogenase (LDH) (OR: 0.607, 95% CI: 0.444–0.831), presence of bone marrow involvement (OR: 2.127, 95% CI: 1.154–3.921) together with reduced complete remission (CR) rate (OR: 0.478, 95% CI: 0.345–0.662).

The results suggest that survivin could be a useful prognostic biomarker, and a promising target for DLBCL therapeutic intervention. Considering limited HR data adjusted for standard prognostic variables could be retrieved, future high-quality studies will be needed in evaluating the independent prognostic value of survivin expression in DLBCL.

## Abstract 2

Supplemental Digital Content is available in the text

## INTRODUCTION

Non-Hodgkin lymphoma is one of the most prevalent malignancies and a leading cause of cancer-related death worldwide. Diffuse large B-cell lymphoma (DLBCL), which is the most common type of aggressive non-Hodgkin lymphoma with increasing incidence, is biologically and clinically heterogeneous malignancy of mature B cells.^1^ In recent years, a growing body of knowledge on the biology of DLBCL has allowed several confounding clinicopathological parameters to be widely applied, such as Ann Arbor stage and International Prognosis Index (IPI) score.^2^ However, existing prognostic parameters are insufficient in present clinical practice. For instance, the IPI score is considered as the current standard prognostic system for the risk stratification of DLBCL. However, heterogeneity in survival is pointed to exist among the patients within the same IPI risk group. Recognizing the biological heterogeneity and the genetic expression profiles, several studies suggested that IPI score might not fully predict the outcome of patients with DLBCL.^3–6^ Therefore, identifying the precisely molecular survival predictors is in unmet clinical needs.^7^ Accordingly, it is valuable and urgent to identify effective biomarkers stratifying patients groups, thus formulating individual therapeutic strategies and improving patients’ survival.

Apoptosis involved in the pathophysiological process of malignant diseases is regulated by 2 families of proteins: the B-cell leukemia/lymphoma 2 family and the inhibitor of apoptosis protein (IAP) family. At 16.5 kDa and of 142 amino acids, survivin, also named as baculoviral IAP repeat containing 5 (BIRC 5), is the smallest and a unique member of IAP family, comprising of antiapoptotic molecules.^8^ It was first identified by Ambrosini et al^8^ from hybridization screening of a human P1 genomic library with the cDNA of effector cell protease receptor/1 in 1997. Accumulating evidence has confirmed the bifunction of survivin in apoptosis inhibition and mitosis regulation. It was demonstrated to inhibit apoptosis by binding specifically to the terminal effector cell death proteases, caspase-3 and -7.^9^ Additionally, it presents a mitosis-regulated pattern of expression during the G2/M phase of the cell cycle.^10^ Intriguingly, survivin was barely detectable in terminally differentiated normal tissues, but it was ubiquitously present in the embryonic tissues.^3^ It was recognized as the 4th most highly expressed protein in human cancer tissue based on data from a large analysis of human transcripts.^6^ Moreover, it was also reported to predict poor outcome in a broad spectrum of solid tumors and various hematological malignances.^12–15^

However, with regard to DLBCL, the prognostic value of survivin expression is indefinite and conflicting. Several previous studies have confirmed that survivin is an independent prognostic indicator in DLBCL.^16–18^ Conversely, Mitrović et al^19^ and Liu et al^20^ illustrated that survivin expression was prognostically irrelevant. This conflict may result from population selection, relatively small sample size, various cut-off levels, and follow-up periods. Thus, to gain a better insight on the prognostic and clinicopathological value of survivin expression in DLBCL, we conducted this meta-analysis of eligible published literature, and systematically evaluated correlation of survivin expression with patients’ clinical outcome, clinicopathogical parameters, and patients’ complete remission (CR) rate which is a crucial indicator to reflect treatment response.

## METHODS

### Search Strategy

A literature search was carried out by using Medline, Embase, Scopus, CNKI, and Wanfang databases up to November 30, 2014. There were no limitations in origin and languages. Search terms were subjected to the following: “survivin,” “baculoviral inhibitor of apoptosis repeat containing 5” or “BIRC5,” “Diffuse large B-cell lymphoma [MeSH],” “expression,” “prognosis” or “overall survival” (OS), etc. All references in retrieved articles were also manually screened to identify additional pertinent studies.

### Selection Criteria

Two investigators independently selected eligible studies. Discrepancies in data extraction were resolved by consensus, referring back to the original article. Inclusion criteria were as follows:

1. All patients were confirmed the diagnosis with DLBCL by a complete history and physical examination, blood morphology and chemistry test, bone marrow biopsy, computed tomography of the chest, and abdomen.
2. Studies focusing on the correlation of survivin expression with survival, clinicopathological characteristics, and CR rate in DLBCL patients. Among this, clinicopathological parameters should comprise of age, gender, clinical stage, B symptoms, Eastern Coorperative Oncology Group performance status, lactic dehydrogenase (LDH) concentration, metastasis to extra nodal sites, and immunosubtypes. Immunosubtypes refer to germinal center like (GCB) subtypes and non-germinal center like (non-GCB) subtypes.
3. Survivin expression model was evaluated by immunohistochemistry (IHC).
4. Articles containing sufficient data to allow the estimation of the value of hazard ratio (HR)/odds ratio (OR) and 95% confidence interval (95% CI) between survivin expression and the survival status, clinicopathological indicators, and CR rate.
5. The number of cases in included studies should be higher than 40.
6. As for the duplicate articles, only the most integrated with the longest follow-up period and/or the recently published one was enrolled.

Only published studies met all the above inclusion requirements were finally included in our meta-analysis. Thus, reviews, case reports, laboratory articles, or letters without key data to calculate OR on clinicopathological features or log hazard ratio (log HR) on survival outcome were excluded.

### Quality Assessment

Quality assessment was conducted for eligible studies by 2 independent reviewers by reading and scoring each publication according to the Newcastle–Ottawa Scale (NOS) Criteria.^21^ This scale evaluates 3 broad perspectives of methodology: subject selection 0 to 4, comparability of subject 0 to 2, and clinical outcome 0 to 3. Total NOS scores range from 0 to 9, and a score ≥7 indicates a good quality. Studies with scores lower than 4 were also excluded in the meta-analysis. Both investigators compared their calculated scores and, if necessary, achieved a consensus score for each category during a meeting.

### Data Extraction

The following data were collected by 2 reviewers independently using a purpose-designed form: the first author's name, publication year, country of the population studied, histology, number of cases and controls, age, study method of protein expression, gender composition, expression level, cut-off level, follow-up period, HR (95% CI) of survival, clinicopathological data, CR rate, and treatment regimens. Any disagreements were resolved by consulting another reviewer.

### Data Synthesis and Analyses

To assess the prognostic significance of survivin expression in patients with DLBCL, pooled HRs and their corresponding 95% CI of OS and event-free survival/disease-free survival (EFS/DFS) were counted. Among our 12 included studies with survival information, we have direct access to adjusted HR data from Adida et al.^16^ In their study, multivariate analysis identified survivin expression as an independent predictive parameter on survival (HR: 1.60, 95% CI: 1.1–2.3) after being adjusted by IPI, performance status, clinical stage, and LDH (lactate dehydrogenase). Meanwhile, with regard to the other 11 studies,^17–18,26–28,30–34,36^ we extrapolated unadjusted values from Kaplan–Meier curves by using software Engauge Digitizer (version 4.1, http://digitizer.sourceforge.net/), and further calculated in methods introduced by Tierney et al^22^ and Parmar et al.^23^

The association between survivin positive expression and clinicopathological parameters and CR (CR versus non-CR) was expressed as OR. Clinicopathological parameters include age (≤60 versus >60), gender (male versus female), clinical stage (stage I + II versus stage III + IV), IPI score (score 0–2 versus score 3–5), B symptoms (Yes versus No), performance status (0–1 versus 1+), serum LDH level (normal/decrease versus increase), extra nodal sites (0–1 versus 1), bone marrow involvement (Yes versus No), and immunosubtypes (GCB versus non-GCB).

By convention, an observed HR > 1 implies a worse survival prognosis for patients with survivin expression. Whereas in this meta-analysis, an observed OR < 1 indicates more probability with positive survivin expression for age above 60, female patients, advanced clinical stage (III + IV), higher IPI score (3–5), absence of B symptoms, performance status above 1, increased LDH level, extra nodal sites above 1, non-GCB immunosubtypes, absence of bone marrow involvement, and reduced CR rate. Furthermore, the effects of survivin expression on survival, clinicopathological features, and CR rate were considered as statistically significant at *P* < 0.05 level, together with the corresponding 95% CI of pooled HR not overlapping 1.

To assess heterogeneity among the studies, we adopted the Chi-squared test and Q test. If heterogeneity was significant, which means *P* < 0.1 or Inconsistency Index (I^2^) >50%, a random effect model with a larger CI and a more conservative standard error, was performed. Otherwise, a fixed effect model was chosen. Begg, Egger linear regression tests, and funnel plots were applied to assess the potential publication bias, and *P* < 0.05 was considered as statistically significant.^19^ Moreover, sensitivity analyses were performed to examine the stability of the pooled studies. All statistical calculations were performed using STATA software (version 12.0, Stata Corporation, College Station, TX).

## RESULTS

### Search Results and Characteristics of Studies

**FIGURE 1:**
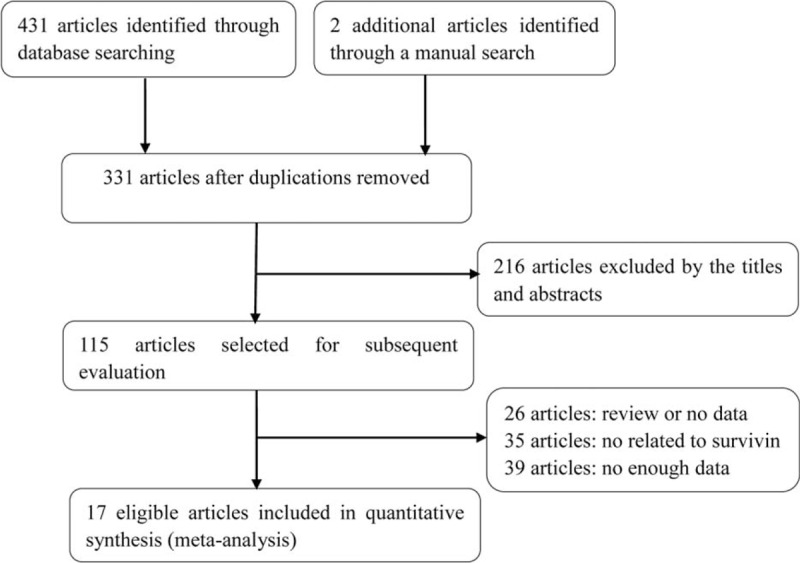
Flow chart of literature search and articles selection.

Detailed articles’ retrieval steps were shown in Figure 1. Initially, a total number of 433 articles were identified. In terms of the titles and abstracts, 216 articles not consistent with inclusion criteria were excluded. And then, the remaining 115 articles went through further evaluation, among which 26 articles were excluded owing to subject of review or no data, 35 for no relation to survivin, and 39 for insufficient data. Eventually, 17 articles^16–21,25–36^ met the selection criteria for quantitative data analysis.

The general characteristics of all 17 studies were summarized in Table 1. A total number of 1352 patients were enrolled in the included studies published between 2000 and 2013. Ten studies originated from China, 1 each from Egypt, Serbia, Croatia, Korea, Turkey, Japan, and America. The percentage of positive survivin expression varies from 26% to 84.90%. Of 17 studies, 14 studies provided various clinicopathological data, 10 studies offering CR information, and HRs and 95% CIs were obtained from 12 studies. Positive survivin expression was investigated by IHC. Since the cut-off values of survivin-positive expression varied among different studies, here we documented the values according to the original articles.

**TABLE 1:**
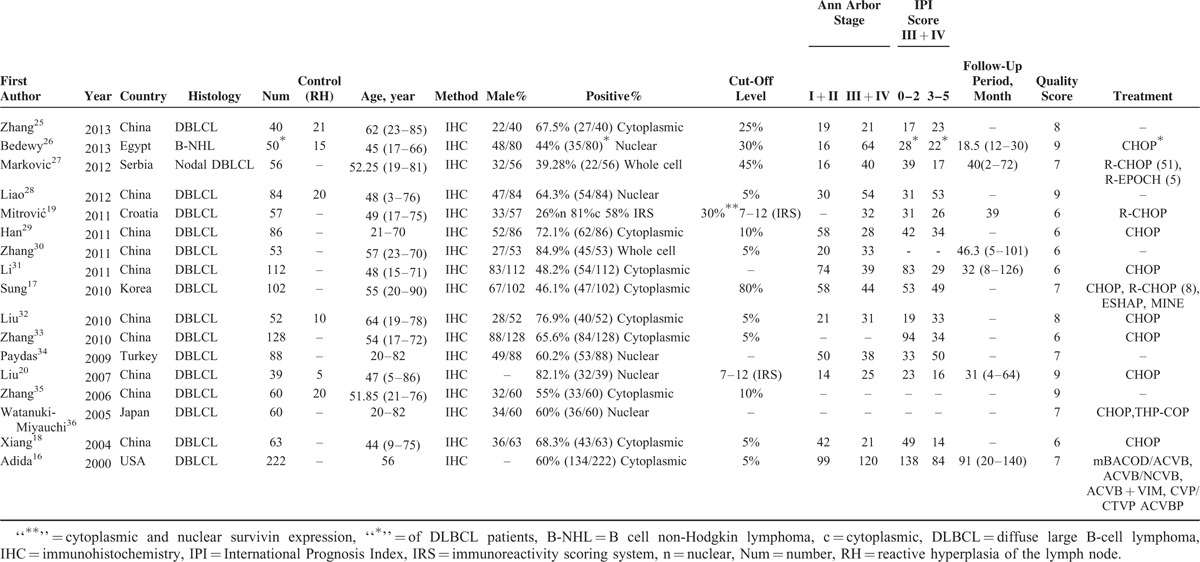
Characteristics in 17 Included Studies

Study quality was evaluated based on the NOS. The quality scores for included articles ranged from 6 to 9, and the median score was 7.24. “High quality” was ranked, when the article was higher than 7.

### Meta-Analysis of Survivin and Patients’ Survival

**FIGURE 2:**
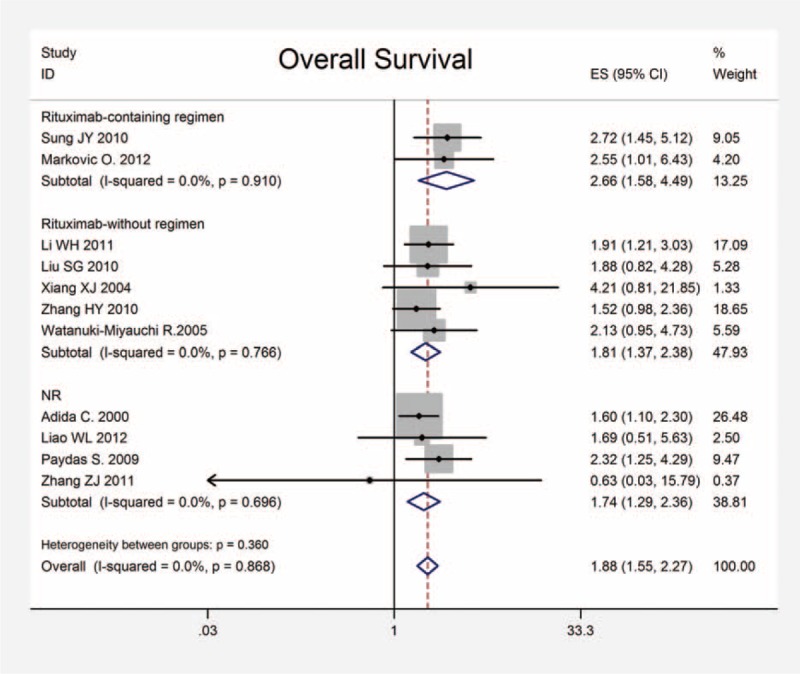
Meta-analysis of the association between survivin expression and OS of patients with DLBCL stratified by the introduction of rituximab regimens. Estimated HR summary for OS is 1.880 (95% CI: 1.550–2.270, *P* < 0.001). CI = confidence interval, DLBCL = diffuse large B-cell lymphoma, HR = hazard ratios, OS = overall survival.

**FIGURE 3:**
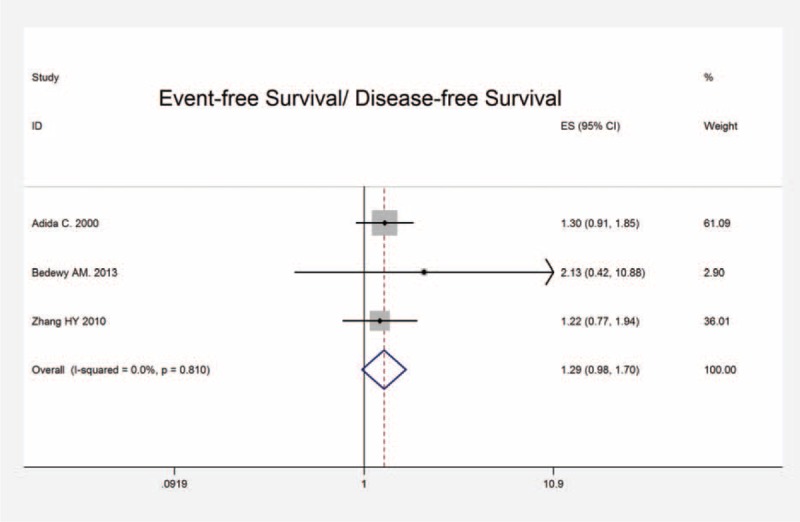
Meta-analysis of the association between survivin expression and EFS/DFS. Estimated HR summary for OS is 1.290 (95% CI: 0.980–1.700, *P* = 0.073). CI = confidence interval, EFS/DFS = event-free survival/disease-free survival, HR = hazard ratios, OS = overall survival.

To assess the prognostic effect of survivin expression in DLBCL, a meta-analysis was performed on HRs of OS and EFS/DFS. The pooled HR and corresponding 95% CI of OS in all 11 studies were 1.880 (95% CI: 1.550–2.270, *P* < 0.001), and no significant heterogeneity was observed (x^2^ = 5.33, *P* = 0.868, I^2^ = 0.0%) (Figure 2). In addition, the combined HRs of the EFS/DFS provided in 3 articles was 1.290 (95% CI: 0.980–1.700, *P* = 0.073) with heterogeneity (x^2^ = 0.42, *P* = 0.810, I^2^ = 0.0%) (Figure 3). Therefore, survivin is indicated to have a significant poor prognostic effect on OS in patients with DLBCL.

Moreover, as the development of rituximab has greatly improved the survival rates in DLBCL, it is of vital clinical significance to estimate the effect of rituximab treatment on the association between survivin expression and the OS. As Figure 2 shows, the combined HRs for rituximab-containing regimen was 2.66 (95% CI: 1.58–4.49, *P* < 0.001), in contrast with 1.81 (95% CI: 1.37–2.38, *P* < 0.001) for rituximab without regimen. The result showed that the introduction of rituximab did not significantly influence the prognostic value of survivin expression in DLBCL (*P* = 0.360) (Figure 2). Besides, we also performed subgroup analyses stratified by survivin staining localization and tissue staining evaluation. Our results indicated that survivin staining localization (cytoplasmic, nuclear, and whole cell) did not make apparent difference in the correlation between survivin and OS (*P* = 0.876). Although evaluating both positive cells percentage and staining intensity was significantly different from evaluating only positive cells in OS (*P* = 0.005).

### Meta-Analysis of Survivin and Patients’ Clinicopathological Variables

**FIGURE 4:**
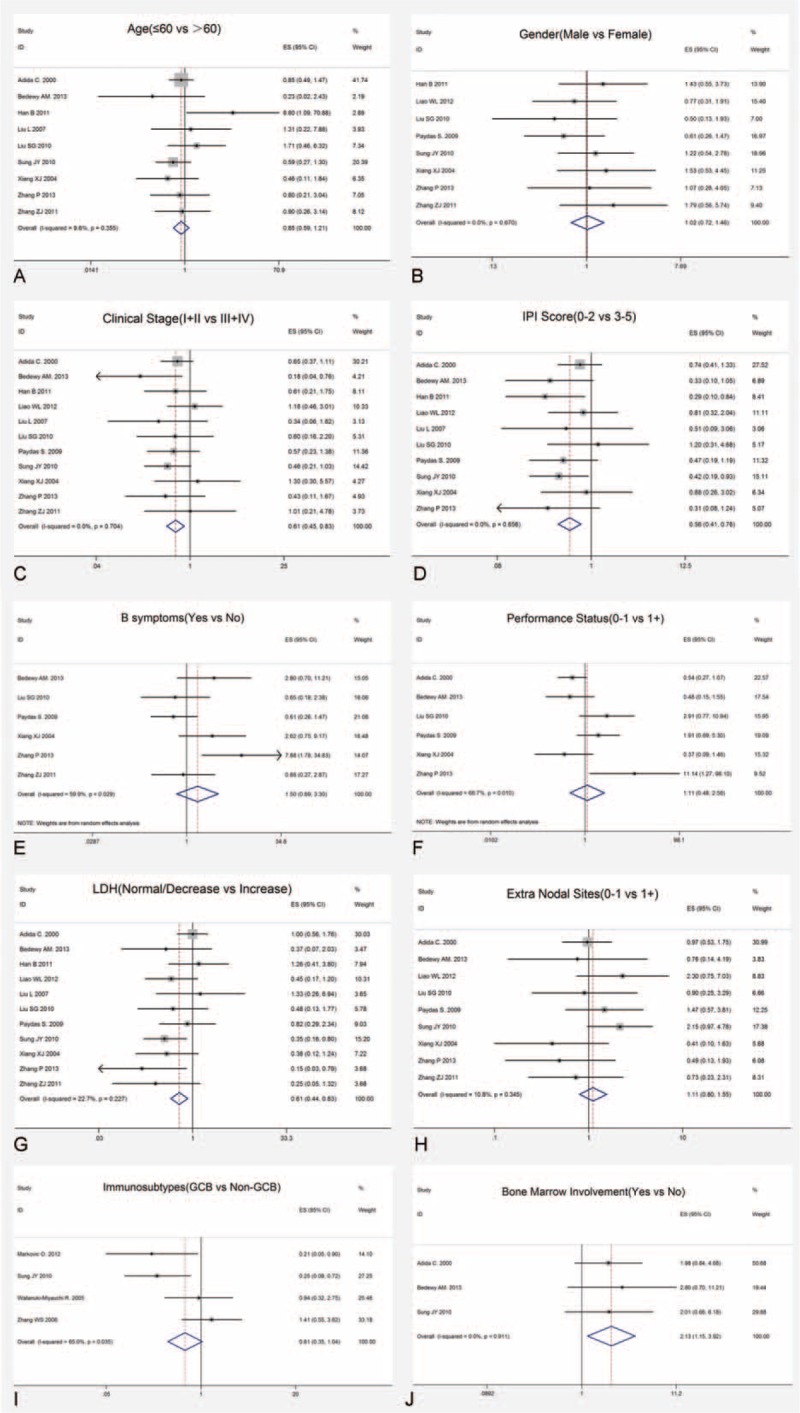
Forrest plots of the relationship between survivin expression and clinicopathological characteristics of DLBCL. (A) Survivin and age, (B) survivin and gender, (C) survivin and clinical stage, (D) survivin and IPI score, (E) survivin and B symptoms, (F) survivin and performance status, (G) survivin and LDH, (H) suvivin and extra nodal sites, (I) survivin and immunosubtypes, and (J) survivin and bone marrow involvement. DLBCL = diffuse large B-cell lymphoma, IPI = International Prognostic Index, LDH = lactic dehydrogenase.

In comprehensive analyses of the role of survivin expression in DLBCL as a biomarker, we investigated the association of survivin overexpression and clinicopathological features. To identify an appropriate statistic model for the combined data, we performed heterogeneity analyses for all clinical-pathological parameters, including age, gender, clinical stage, IPI score, presence of B symptoms, performance status, LDH level, metastasis to extra nodal sites, bone marrow involvement, and immunosubtypes (GCB, non-GCB). Fixed effect models revealed a significant association between survivin expression and advanced clinical stage (stage III + IV) (OR: 0.611, 95% CI: 0.452–0.827, *P* = 0.001), higher IPI score (score 3–5) (OR: 0.559; 95% CI: 0.410–0.761, *P* < 0.001), increased LDH level (OR: 0.607, 95% CI: 0.444–0.831, *P* = 0.002) together with presence of bone marrow involvement (OR: 2.127, 95% CI: 1.154–3.921, *P* = 0.016) (Figure 4). No heterogeneity and publication bias were revealed. However, no association was observed regarding survivin with age (OR: 0.845, 95% CI: 0.593–1.205, *P* = 0.353), gender (OR: 1.002, 95% CI: 0.716–1.461, *P* = 0.903), positive B symptoms (OR: 1.505, 95% CI: 0.686–3.302, *P* = 0.308), performance status (OR: 1.109, 95% CI: 0.480–2.560, *P* = 0.809), extra nodal sites (OR: 1.113, 95% CI: 0.798–1.552, *P* = 0.529), GCB and non-GCB (OR: 0.607, 95% CI: 0.353–1.044, *P* = 0.071). There was no significant heterogeneity identified neither. All the above-suggested survivin expression in DLBCL patients was strongly linked to inferior clinical outcome, which means high grade, high IPI score, increased LDH, and bone marrow involvement.

### Meta-Analysis of Survivin and Patients’ CR Rate

**FIGURE 5:**
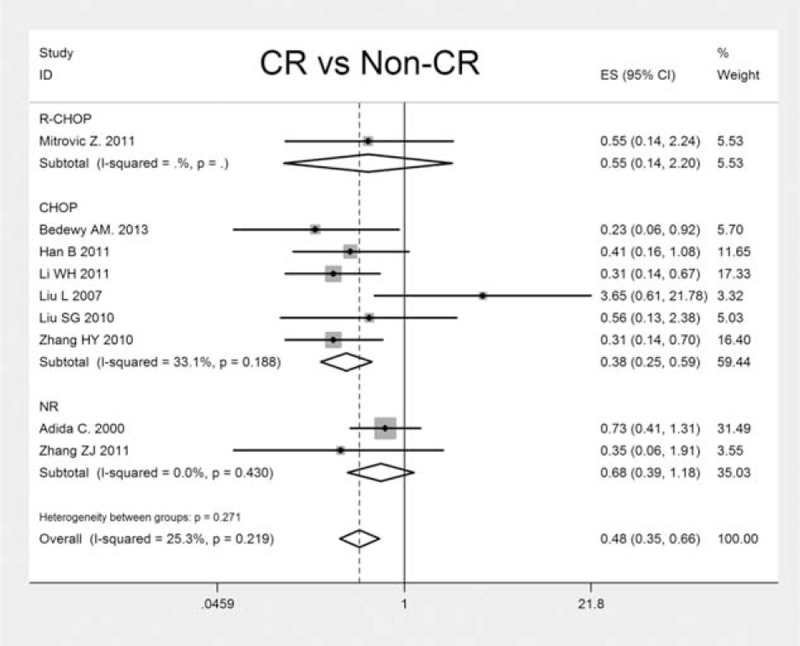
The individual and pooled OR with 95 % CI of survivin expression and CR rate in patients with DLBCL. A fixed effect model revealed an association between survivin and CR rate (CR, non-CR) (*n* = 9, OR: 0.478, 95 % CI: 0.345–0.662; *P* < 0.001). CI = confidence interval, CR = complete remission, DLBCL = diffuse large B-cell lymphoma, OR = odds ratio.

CR rate is a vital indicator for the assessment of prognosis and therapeutic efficacy in patients with DLBCL. In this meta-analysis, 9 eligible studies were included to evaluate the correlation of survivin expression and patients’ CR (Figure 5). The combined OR and 95% CI of patients’ CR were 0.478 (95% CI: 0.345–0.662, *P* < 0.001), and no significant heterogeneity was revealed (x^2^ = 10.71, *P* = 0.219, I^2^ = 25.3%). It suggested that positive survivin expression was in significant association with patients’ reduced CR rate.

Currently, R-CHOP (rituximab plus cyclophosphamide, hydroxydoxorubicin, vincristine, and prednisone) regimen is widely acknowledged as the standard chemotherapy protocol in treating newly diagnosed patients with DLBCL.^37,38^ To further analyze the effect of survivin expression on patients’ CR with different chemotherapy regimens, we stratified the treatments by R-CHOP and CHOP. The result suggested that the introduction of rituximab did not alter the association of survivin expression and patients’ CR significantly (*P* = 0.627). Future studies with larger sample sizes need to be conducted to verify our result.

### Sensitivity Analyses

Sensitivity analyses showed that the pooled HR/ORs were not significantly influenced after omitting any single study and the rest were analyzed, which support the reliability and stability of our results. Figures of sensitivity analyses of random effects meta-analysis estimates and analyses including 10 or more studies were shown in Supplemental Figure 1, http://links.lww.com/MD/A399.

### Publication Bias

In the present meta-analysis, we introduced Begg and Egger regression tests as well as funnel plots to assess publication bias. As is indicated in Table 2, no publication bias was observed statistically for survivin expression with regard to OS, EFS/DFS, clinical-pathological indicators, and patients’ CR. Furthermore, the shape of funnel plots did not reveal obvious evidence of asymmetry, suggesting that no extra publication bias was also observed among studies (figures not shown).

**TABLE 2:**
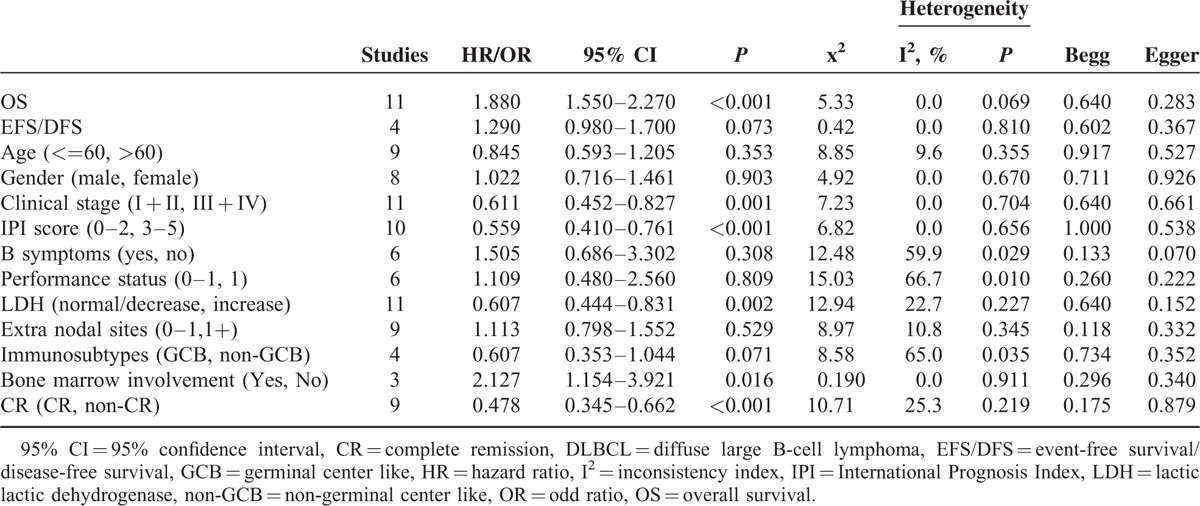
Main Meta-Analysis Results

## DISCUSSION

In view of evidence on high expression of survivin in a myrid of malignancies, survivin was identified as an attractive potential prognostic factor and state-of-art therapeutic target in cancer.^39^ Yet the prognostic and clinicopathological value of survivin are still inconsistent and controversial in DLBCL. Some studies indicated that survivin predicted a poor prognosis in patients with DLBCL.^16–18^ Although Mitrović et al^19^ and Liu et al^20^ pointed that survivin expression was not associated with patients clinical outcomes, and suggested it not to be identified as an useful prognostic marker in DLBCL. Additionally, whether survivin is relevant to clinicopathological parameters and CR rate in patients with DLBCL still need to be clarified. Therefore, we perform this clinically significant meta-analysis trying to settle the remaining conflict and provide evidence on the correlation.

Based on literature selection criteria and NOS quality assessment scale, we finally included 17 eligible studies with 1352 patients. Our study yields important results concerning the actual effect of survivin expression on prognosis, clinicopathology, and therapeutic response of patients with DLBCL. The results showed the pooled HR and 95% CI of OS and EFS/DFS were 1.880 (95% CI: 1.550–2.270, *P* < 0.001) with heterogeneity (x^2^ = 5.33, *P* = 0.868, I^2^ = 0.0%) and 1.290 (95% CI: 0.980–1.700, *P* = 0.073) with heterogeneity (x^2^ = 0.42, *P* = 0.810, I^2^ = 0.0%), respectively, which provided direct evidence that high survivin expression is significantly related to worse OS of patients. Although with regard to clinicopathological parameters, significant associations were revealed between survivin expression and advanced clinical stage (stage III + IV) (OR: 0.611, 95% CI: 0.452–0.827, *P* = 0.001), higher IPI score (score 3–5) (OR: 0.559, 95% CI: 0.410–0.761, *P* < 0.001), increased LDH level (OR: 0.607, 95% CI: 0.444–0.831, *P* = 0.002) along with presence of bone marrow involvement (OR: 2.127, 95% CI: 1.154–3.921, *P* = 0.016). By interacting with cytokines/growth factor, adhesion molecules and proteinases, survivin exerts a critical role in tumor invasion and metastasis,^40^ which may mechanistically further explain why survivin were overexpressed in high grade, invasive DLBCL. Besides, it has been widely acknowledged that elevated LDH is associated with increased likelihood of relapse in DLBCL patients.^41^ Survivin apparent high expression in relapsed patients sheds light on the potential effectiveness of survivin suppressors targeting relapsed DLBCL patients. As for the indicator of patients’ therapeutic response, CR presented direct relationship with survivin expression (OR: 0.478, 95% CI: 0.345–0.662, *P* < 0.001). Accumulating evidence has confirmed that survivin is responsible for chemoresistance in various malignances, which may account for patients’ reduced CR rate in DLBCL.^42^

Sources of heterogeneity in the pooled analyses were explored by Chi-squared test and classic Q statistic test. Random-effects model was utilized in case of potential heterogeneity. Specifically, substantial heterogeneity of the analyses on B symptoms and PS were ascribed to Zhang et al,^25^ and heterogeneity of immunosubtypes was due to Zhang et al.^35^ Zhang et al^25^ attributed to the heterogeneity on limited sample size (40 patients) and different cut-off levels. Moreover, in analysis of association between survivin and immunosubtypes, Zhang et al^35^ was the only study revealing that survivin expresses more in GCB than in non-GCB. Besides, different from other studies, the Maxvision immunohistochemical method it adopted may result in its statistical heterogeneity.

Generally, heterogeneity derives from many aspects. Firstly, there are still no putative criteria to define the positive expression of survivin, which may result in discrepancy. Our subgroup analyses pointed that survivin staining localization did not make apparent difference (*P* = 0.876). Besides, evaluating both positive cells percentage and staining intensity was significantly different from evaluating only positive cells on OS (*P* = 0.005), indicating a potential source of heterogeneity. Furthermore, the definition of cut-off value varied among the studies, which can also produce heterogeneity. Eventually, it is reasonable to generate heterogeneity on HR extrapolation. Despite being undertaken by 2 reviewers, for HRs extracted from the survival curves, inaccuracy is inevitable.

Several limitations need to be pointed out. Above all, among our 12 included studies, only Adida et al^16^ provided adjusted HR information. Insufficient retrievable HR data adjusted for standard prognostic variables might not convincingly guarantee the independent prognostic significance of survivin expression in DLBCL. Besides, although survivin expression in the included studies was all measured by IHC, the detailed methodological factors such as primary antibody and secondary antibody concentrations were not consistent, contributing to certain bias. In addition, population-level data rather than patient-level data were extracted, which limit our ability to test for associations between variables in specific subgroups. What is more, most studies are inclined to report positive outcomes, whereas the studies with negative results are often rejected or less assessable, giving rise to the publication bias.

In conclusion, despite the limitations, our meta-analysis provides robust evidence on the prognostic and clinicopathological value of survivin in DLBCL. It demonstrates a significant correlation between survivin expression with poor prognosis, including worse OS, advanced clinical stage, high IPI score, increased LDH, presence of bone marrow involvement, and reduced CR rate in patients with DLBCL. Furthermore, the direct relationship to patient's inferior outcomes is clinically beneficial in highlighting the application of survivin inhibitors on relapsed/refractory DLBCL patients, which may open a new scenario to cancer-targeted therapy in DLBCL. To verify our results, further multicenter prospective studies with standardized methods, long-term follow-up are needed.
